# *In Vitro* Synergy of Isavuconazole Combined With Colistin Against Common *Candida* Species

**DOI:** 10.3389/fcimb.2022.892893

**Published:** 2022-04-29

**Authors:** Patrick Schwarz, Ilya Nikolskiy, Anne-Laure Bidaud, Frank Sommer, Gert Bange, Eric Dannaoui

**Affiliations:** ^1^ Department of Internal Medicine, Respiratory and Critical Care Medicine, University Hospital Marburg, Marburg, Germany; ^2^ Center for Invasive Mycoses and Antifungals, Faculty of Medicine, Philipps University Marburg, Marburg, Germany; ^3^ Center for Synthetic Microbiology (SYNMIKRO), Department of Chemistry, Philipps University Marburg, Marburg, Germany; ^4^ Unité de Parasitologie-Mycologie, Hôpital Européen Georges-Pompidou, Paris, France; ^5^ Department of Microbiology, University Hospital Marburg, Marburg, Germany; ^6^ Max Planck Fellow Group, Molecular Physiology of Microbes, Max Planck Institute for Terrestrial Microbiology, Marburg, Germany; ^7^ Dynamyc Research Group (EA 7380), Faculté de Médecine de Créteil, Université Paris-Est-Créteil-Val-de-Marne, Créteil, France; ^8^ Faculté de Médecine, Université de Paris, Paris, France

**Keywords:** antifungal combination, *Candida*, colistin, EUCAST, *in vitro*, isavuconazole

## Abstract

Interactions of isavuconazole and colistin were evaluated against 57 common *Candida* strains belonging to the species *Candida albicans* (n = 10), *Candida glabrata* (n = 10), *Candida kefyr* (n = 8), *Candida krusei* (n = 10), *Candida parapsilosis* (n = 9), and *Candida tropicalis* (n = 10) by a broth microdilution checkerboard technique based on the European Committee on Antimicrobial Susceptibility Testing (EUCAST) reference methodology for antifungal susceptibility testing. Results were analyzed with the fractional inhibitory concentration index and by the response surface analysis. Interpretation by the fractional inhibitory concentration index showed synergy for 50%, 80%, 90%, and 90% of the *C. kefyr*, *C. krusei*, *C. glabrata*, and *C. tropicalis* strains, respectively. Combination of isavuconazole with colistin against *C. albicans* and *C. parapsilosis* exhibited only indifference for 100% and 90% of the strains, respectively. The results were confirmed by response surface analysis for all species except for *C. glabrata*, for which an indifferent interaction was found for the majority of strains. Antagonistic interaction was never seen regardless of the interpretation model was used.

## Introduction

Candidemia is a severe and life-threatening infection caused by different *Candida* species. In Germany, candidemia ranks as the 6th most common bloodstream infection after *Escherichia coli*, *Staphylococcus aureus*, coagulase-negative staphylococci, *Klebsiella pneumoniae*, and enterococci (Schöneweck et al., 2021). From the bloodstream, *Candida* can disseminate to multiple organs involving most commonly the liver, spleen, kidney, myocardium, or eyes and less frequently the brain (Pilmis et al., 2017). Invasive candidiasis is associated with a high mortality rate of about 40%. While many different *Candida* species can be responsible for invasive diseases, 95% of the infections are caused by the five species *Candida albicans*, *Candida glabrata*, *Candida krusei*, *Candida parapsilosis*, and *Candida tropicalis* (Yapar, 2014). Although *C. albicans* is still responsible for the majority of infections in Germany (Mohr et al., 2020; Schroeder et al., 2020), non-*albicans Candida* species can represent up to 60% of the cases of candidemia in other parts of the world like France (Vannini et al., 2022), Italy (Montagna et al., 2013), or the United States (Pfaller et al., 2012). Compiled data from the ARTEMIS DISK registry including data from 147 medical centers in 41 countries all over the world for the years 1997–2007 indicated that despite a decrease in frequency, *C. albicans* remained the most frequent species worldwide, while the frequency of *C. glabrata* and *C. krusei* was stable, and the frequency of *C. parapsilosis* and *C. tropicalis* increased (Pfaller et al., 2010). Echinocandins are recommended as first-line therapies for invasive candidiasis, and azoles can be used, as step-down therapies (Cornely et al., 2012), but decreased susceptibility and even resistance to antifungals can occur. Decreased susceptibility or drug resistance in *Candida* species can be intrinsic, like decreased caspofungin susceptibility in *C. parapsilosis* (Pfaller et al., 2006) or fluconazole resistance in *C. krusei* (Orozco et al., 1998), or can be acquired like echinocandin resistance in *C. glabrata* (Aldejohann et al., 2021). The high mortality rate, the shift towards more difficult to treat *Candida* species, and the lack of efficacy in monotherapy for some difficult-to-treat infections make alternative approaches necessary (Vitale, 2021). It has been shown *in vitro* (Schwarz et al., 2007), *in vivo* (Schwarz et al., 2006b), and in patients (Day et al., 2013) that a combination of two antifungals can increase their potency and decrease resistance. However, against invasive candidiasis, no favorable combination has been found (Johnson et al., 2004). Apart from the combination of two antifungals, combinations of antifungal and other molecules can also lead to favorable interactions (Schwarz et al., 2019). The antibiotic colistin has shown *in vitro* synergy in combination with amphotericin B against *Candida* species (Schwarz et al., 2022), but routine use in critically ill patients is limited due to the nephrotoxicity of the molecules. Isavuconazole is a broad-spectrum azole with favorable tolerability (Ledoux et al., 2018), which makes it an interesting partner for combinations of molecules. *In vitro* combination of isavuconazole with colistin has been shown to be synergistic against *Aspergillus nidulans*, *Aspergillus niger* (Schwarz et al., 2020b), and *Candida auris* (Schwarz et al., 2020a) and could thus be also an interesting combination against *Candida* species. We therefore explored the combination of isavuconazole with colistin against common *Candida* species by a checkerboard technique based on the European Committee on Antimicrobial Susceptibility Testing (EUCAST) methodology for antifungal susceptibility testing.

## Materials and Methods

### Strains

In this study, a total of 57 clinical *Candida* strains belonging to 6 common *Candida* species were included. *Candida* species included 10 C*. albicans*, 10 C*. glabrata* (teleomorph in the *Nakaseomyces* clade), 8 *Candida kefyr* (teleomorph *Kluyveromyces marxianus*), 10 C*. krusei* (teleomorph *Pichia kudriavzevii*), 9 C*. parapsilosis*, and 10 C*. tropicalis*. *Candida* strains were mainly obtained from the Department of Microbiology of the University Hospital Marburg (n = 53). Additional isolates (n = 4) belonged to the collections of the American Type Culture Collection (ATCC) and the Deutsche Sammlung von Mikroorganismen und Zellkulturen (DSM). The complete Internal Transcribed Spacer (ITS)1-5.8S-ITS2 region of the non-collection strains was sequenced as described elsewhere (Schwarz et al., 2006a) to obtain molecular identification of the strains to the species level. Sequences were deposited at GenBank under the accession numbers OL351325 to OL351356 (Schwarz et al., 2022) and under OM859334 to OM859357. Only *C. krusei* strain U2106778 was identified by matrix-assisted laser desorption ionization–time of flight (MALDI-TOF) due to ITS region sequence heterogeneity (Zhao et al., 2015). *C. krusei* ATCC 6258 and *C. parapsilosis* ATCC 22019 were used as quality controls in each batch of microplates.

### Drugs

Isavuconazole (Pfizer, Berlin, Germany) stock solution was prepared at 3,200 µg/ml in dimethyl sulfoxide (DMSO). A stock solution of colistin (Merck, Darmstadt, Germany) at 12,800 µg/ml was prepared in sterile, distilled water. Stock solutions were kept at −25°C until use.

### Medium Preparation

For this study, Roswell Park Memorial Institute 1640 (RPMI) medium (with l-glutamine, with pH indicator, but without bicarbonate) (Merck) prepared in double strength was used as the test medium. It contained 2% (w/v) of d-glucose and was buffered with 3-(*N*-morpholino)propanesulfonic acid (Merck) at a final concentration of 0.165 mol/L. The final pH of 7.0 was adjusted with 2 molar NaOH. The medium was sterilized through a 0.22-µm pore size filter by vacuum filtration (Merck).

### Microplate Preparation

An antifungal susceptibility testing protocol, modified for broth microdilution checkerboard procedures, based on the EUCAST guidelines was used in this study (Arendrup et al., 2020). Combination experiments were carried out in Nunclon™ delta surface 96-well microtiter plates for adherent cells (Thermo Fisher Scientific, Darmstadt, Germany). The combination of isavuconazole with colistin was studied on a two-dimensional checkerboard (Bidaud et al., 2021). Twofold serial dilutions of each drug were done in the double strength test medium. Final concentrations for isavuconazole ranged from 0.0001 to 0.03 µg/ml for *C. albicans*, *C. kefyr*, *C. parapsilosis*, and *C. tropicalis* or 0.002–0.5 µg/ml for *C. glabrata* and *C. krusei*. Colistin concentrations ranged from 1 to 64 µg/ml for all tested species. Before the addition of the inoculum, each well contained 100 µl of double-strength RPMI medium with 1% (v/v) of DMSO.

### Inoculum Preparation and Inoculation of Microplates

Before the experiments, *Candida* strains were cultured on Sabouraud dextrose agar slants supplemented with chloramphenicol and gentamicin (Bio-Rad Laboratories, Feldkirchen, Germany) at 35°C and 95% humidity for 24 h. By using inoculation loops, fungi were transferred from the agar slants to sterile tubes containing pure water. After the cells were counted in a hemocytometer, the suspensions were adjusted to the final inoculum size of 2 × 10^5^ colony forming units (CFU)/ml. One hundred microliters of the inoculum was distributed into each well of the microplates using Eppendorf Xplorer plus (Eppendorf, Hamburg, Germany) electric multichannel pipettes. After incubation at 35°C and 95% humidity for 24 h, optical densities were read spectrophotometrically at a wavelength of 530 nm using a MultiSkan FC spectrometer (Thermo Fisher Scientific). Before the reading, microplates were shaken for 2 min at 1,100 rpm with a PMS-1000 Microplate Shaker (Grant Instruments, Shepreth, UK). Before the analysis of the results, the optical density values of a blank plate, each well inoculated with 100 µl of sterile distilled water and incubated under the same conditions as mentioned above, were subtracted from the values of the microplates inoculated with yeast cells. The final inoculum was further diluted at 1:10, and 50 µl were spread once on Sabouraud dextrose agar plates with a sterile Drigalski spatula. After 24 h of incubation, CFU were counted to ensure inoculum size. Combination experiments were run in duplicate.

### Interpretation of the Results by Fractional Inhibitory Concentration Index

Optical density values from the microplates were transformed into a percentage of growth compared to the growth control. Fifty percent of inhibition was chosen as an endpoint for the determination of the minimum inhibitory concentrations (MICs) alone for both drugs and in combination. High off-scale MICs were converted to the next log_2_ dilution. If the lowest fractional inhibitory concentration index (FICI) on the microplate was ≤0.5 or >0.5 to 4, synergy or indifference was assumed, respectively. For FICIs higher than 4.0, antagonism was concluded (Odds, 2003).

### Interpretation of the Results by Response Surface Analysis

Compared to the FICI, response surface analysis allows the evaluation of drug interactions without using an inhibition endpoint and is therefore independent of MICs. It enables the determination (and visualization) of the interaction for all tested concentrations and not only for the MICs in combination. Based on the growth rates in the wells of the molecules alone (in this study isavuconazole and colistin), dose–response curves for the drugs alone are generated. According to the chosen theoretical model, using the dose–response curves of the two drugs alone, an indifferent dose–response surface is calculated. In this study, the Bliss independence model was chosen, which is based on the hypothesis that drugs act independently from each other. To determine the interaction of the molecules, the (experimentally) observed combination dose–response surface is compared to the predicted (calculated) indifferent dose–response surface. A combination effect is defined as synergistic if the observed effect lies below the predicted indifferent dose–response surface (corresponds to less growth on the microplate and a greater effect of the combination) and antagonistic when the observed effect lies above (corresponds to more growth on the microplate and a weaker effect of the combination). To quantitatively assess the interaction of the drugs, the SUM-SYN-ANT metric is calculated, which is defined as the sum of all effects greater than the predicted indifferent effect (SYN-SUM), minus the sum of all effects weaker than the predicted indifferent effect (ANT-SUM). The intrinsic variability of the broth microdilution technique necessitates the definition of a threshold, for which the interaction of the two drugs is defined as indifferent. This threshold is determined experimentally by combining the active molecules with themselves. Therefore, the combination of isavuconazole with itself was tested on the two-dimensional checkerboard with twofold serial dilutions as described above. The highest concentration of isavuconazole was 0.12 µg/ml in the x- and y-axes. For the determination of the threshold, *C. krusei* ATCC 6258 was tested in triplicate on these plates. Based on the results of the experimental plates, synergy was assumed when the SUM-SYN-ANT was ≥56.0%, and antagonism was assumed when ≤−56.0%. Between −56.0% and 56.0%, indifference was concluded.

To determine the SUM-SYN-ANT metric of the different tested strains, the results of both runs were combined. All calculations were done by the Combenefit software (http://sourceforge.net/projects/combenefit/) (Di Veroli et al., 2016).

## Results

**Figure 1:**
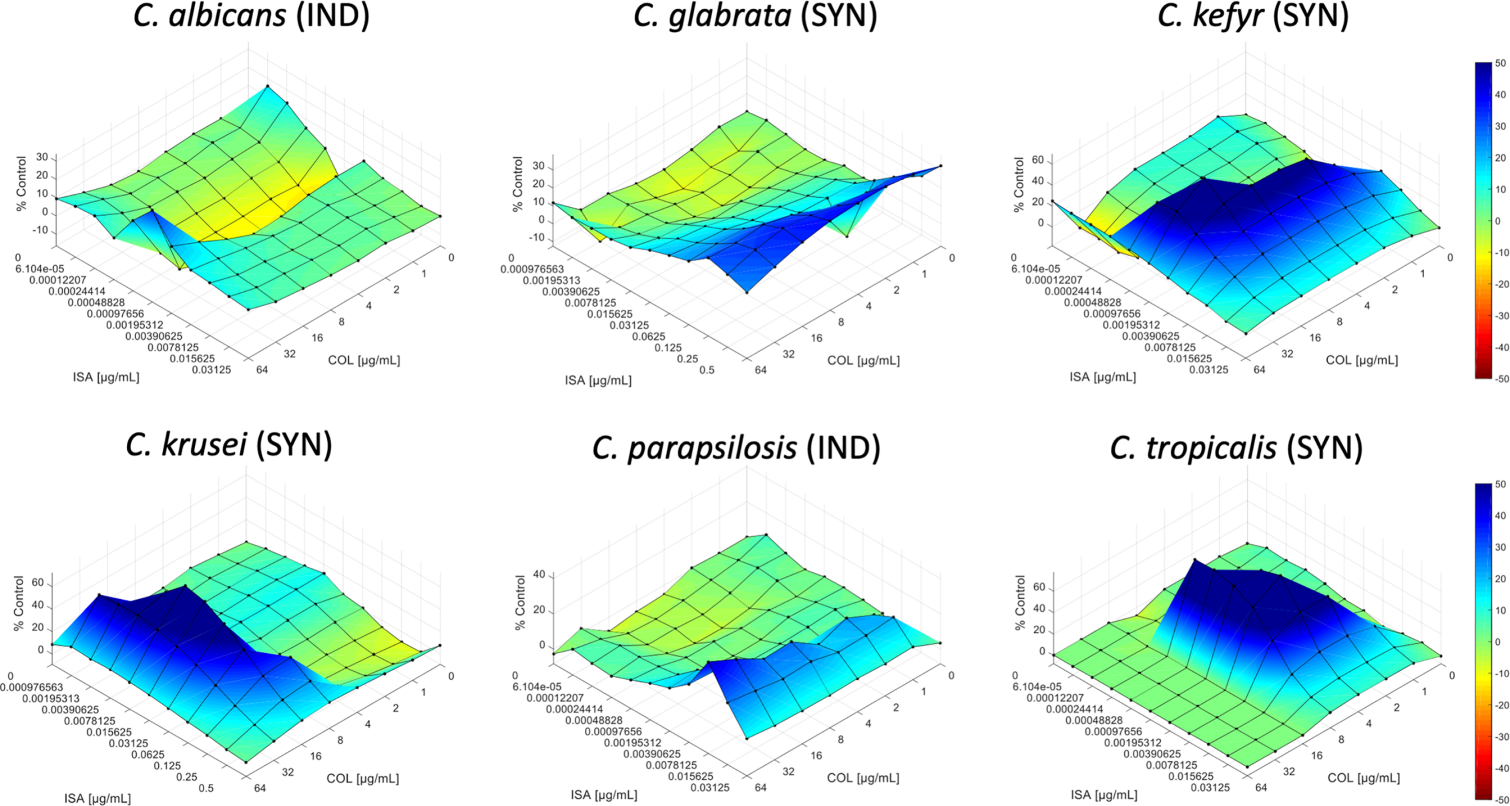
Synergy distribution for the combination of isavuconazole with colistin against *Candida albicans* V2105529, *Candida glabrata* U2106602, *Candida kefyr* V2108462, *Candida krusei* N2102435, *Candida parapsilosis* V2105056, and *Candida tropicalis* N2102715. The mode of interaction was defined based on the SUM-SYN-ANT metric. IND, indifference; SYN, synergy.

The interactions of isavuconazole with colistin were evaluated by checkerboard and interpretation of the results by FICI or by response surface analysis against strains from six common *Candida* species as presented in **Table 1**. A summary of the results is presented in **Table 2**. **Figure 1** shows the synergy distributions for the combination of isavuconazole with colistin against a representative isolate of each tested species.

**Table 1:** Interaction of isavuconazole with colistin against common *Candida* species by checkerboard and interpretation by fractional inhibitory concentration index and response surface analysis.

Species	Collection number	Checkerboard MICs (µg/ml)					Response surface analysis	
		ISA	COL	ISA/COL	FICI	INTPN	ΣSYN-ANT (ΣSYN; ΣANT)	INTPN
*Candida albicans*	V2105126	0.002	128	0.001/16	0.625	IND	−0.76 (21.00; −21.76)	IND
*C. albicans*	N2101578	0.0005	128	0.001/64	0.75	IND	−26.21 (15.38; −41.59)	IND
*C. albicans*	V2105568	0.002	128	0.00006/64	0.625	IND	30.84 (36.97; −6.13)	IND
*C. albicans*	N2101577	0.002	128	0.001/64	1	IND	13.88 (29.36; −15.48)	IND
*C. albicans*	V2105825iso3	0.002	128	0.001/32	0.75	IND	31.53 (33.87; −2.34)	IND
*C. albicans*	ATCC 14053	0.002	128	0.0002/64	0.625	IND	−18.30 (22.32; −40.62)	IND
*C. albicans*	V2105529	0.002	128	0.125/32	0.75	IND	5.75 (19.05; −13.30)	IND
*C. albicans*	V2106139	0.004	128	0.125/2	0.5156	IND	22.27 (28.42: −6.15)	IND
*C. albicans*	V2106041	0.002	64	0.06/1	0.5156	IND	−0.48 (29.70; −0.48)	IND
*C. albicans*	V2106305	0.004	128	0.03/16	0.625	IND	−3.57 (21.74; −25.31)	IND
*Candida glabrata*	V2105272	0.5	128	0.125/2	0.2656	SYN	39.63 (41.86; −2.23)	IND
*C. glabrata*	V2105282	0.5	128	0.125/8	0.3125	SYN	51.39 (51.73; −0.34)	IND
*C. glabrata*	N2101711	0.125	128	0.06/2	0.5156	IND	25.15 (31.05: −5.90)	IND
*C. glabrata*	V2105636	0.125	128	0.003/16	0.375	SYN	12.57 (25.00; −12.43)	IND
*C. glabrata*	DSM 70614	0.125	128	0.008/32	0.3125	SYN	33.03 (41.54; −8.51)	IND
*C. glabrata*	U2105834	0.125	128	0.03/16	0.375	SYN	52.37 (53.11; −0.74)	IND
*C. glabrata*	V2105576	0.125	128	0.03/32	0.5	SYN	33.35 (38.12; −4.77)	IND
*C. glabrata*	N2102530	0.125	128	0.03/8	0.3125	SYN	43.61 (47.42; −3.81)	IND
*C. glabrata*	U2106503	0.125	128	0.03/8	0.3125	SYN	52.90 (53.27; −0.37)	IND
*C. glabrata*	U2106602	0.25	64	0.03/8	0.25	SYN	60.21 (65.28; −5.07)	SYN
*Candida krusei*	V2105825iso4	0.125	64	0.016/16	0.375	SYN	37.13 (48.62; −11.49)	IND
*C. krusei*	V2105866	0.125	64	0.016/16	0.375	SYN	62.16 (71.23; −9.07)	SYN
*C. krusei*	V2106177	0.25	64	0.06/16	0.5	SYN	75.43 (77.34; −1.91)	SYN
*C. krusei*	V2105920	0.25	64	0.06/16	0.5	SYN	122.67 (123.03; −0.36)	SYN
*C. krusei*	ATCC 6258	0.03	16	0.016/2	0.625	IND	13.01 (23.66; −10.65)	IND
*C. krusei*	N2102290	0.125	128	0.03/32	0.5	SYN	12.20 (28.93; 16.73)	IND
*C. krusei*	N2102435	0.125	64	0.004/16	0.2813	SYN	90.28 (92.24; −1.96)	SYN
*C. krusei*	U2106649	0.125	64	0.03/16	0.5	SYN	53.12 (55.80; −2.68)	IND
*C. krusei*	U2106778	0.125	64	0.03/2	0.5313	IND	14.33 (54.69; −40.36)	IND
*C. krusei*	V2108462iso101	0.25	32	0.03/8	0.5	IND	76.01 (76.09; −0.08)	SYN
*Candida parapsilosis*	V2105056	0.016	128	0.004/4	0.2813	SYN	43.71 (46.49; −2.78	IND
*C. parapsilosis*	V2105223	0.016	128	0.004/1	0.2578	SYN	55.49 (65.12; −9.63)	IND
*C. parapsilosis*	B2107379	0.008	128	0.004/1	0.5078	IND	28.56 (29.81; −1.25)	IND
*C. parapsilosis*	ATCC 22019	0.03	64	0.008/8	0.375	SYN	7.38 (36.44; −29.06)	IND
*C. parapsilosis*	U2106978	0.016	128	0.008/1	0.5078	IND	−14.61 (4.96; −19.57)	IND
*C. parapsilosis*	V2111362	0.008	128	0.004/2	0.5156	IND	38.05 (38.26; −0.21)	IND
*C. parapsilosis*	V2111422	0.004	64	0.002/2	0.5313	IND	22.59 (32.49; −9.90)	IND
*C. parapsilosis*	V2113147	0.008	128	0.004/64	1	IND	−48.79 (8.98; −57.77)	IND
*C. parapsilosis*	V2113210	0.016	128	0.008/2	0.5156	IND	34.82 (43.88; −9.06)	IND
*Candida tropicalis*	V2105128	0.008	16	0.002/16	0.5	SYN	12.31 (33.75, −21.44)	IND
*C. tropicalis*	V2105245	0.008	16	0.002/16	0.5	SYN	23.42 (29.74; −6.32)	IND
*C. tropicalis*	V2105598	0.008	16	0.002/16	0.5	SYN	88.36 (88.39; −0.03)	SYN
*C. tropicalis*	B1907975	0.008	32	0.002/32	0.375	SYN	48.49 (59.88; −11.39)	IND
*C. tropicalis*	V2106298	0.008	32	0.002/32	0.5	SYN	48.95 (74.91; −25.96)	IND
*C. tropicalis*	U2106694	0.06	16	0.016/4	0.5	SYN	47.39 (47.62; −0.23)	IND
*C. tropicalis*	U2107090	0.06	32	0.016/4	0.375	SYN	66.67 (67.74; −1.06)	SYN
*C. tropicalis*	N2102715	0.008	16	0.002/2	0.375	SYN	75.98 (76.32; −0.34)	SYN
*C. tropicalis*	V2108236	0.008	16	0.002/4	0.5	SYN	65.93 (66.36; −0.43)	SNY
*C. tropicalis*	V2108424	0.008	16	0.0005/8	0.5625	IND	65.12 (66.25; −1.13)	SYN
*Candida kefyr*	V2105566	0.004	32	0.002/2	0.5625	IND	5.84 (30.25; −24.41)	IND
*C. kefyr*	V2106126	0.004	64	0.001/16	0.5	SYN	100.81 (100.81; −0.0)	SYN
*C. kefyr*	N2101899	0.001	16	0.0005/4	0.75	IND	−0.91 (9.10; −10.01)	IND
*C. kefyr*	N2102541	0.002	8	0.001/8	0.5625	IND	69.86 (70.78; −0.92)	SYN
*C. kefyr*	V2107273	0.06	4	0.016/4	0.5	SYN	15.56 (35.50; −19.94)	IND
*C. kefyr*	V2107293	0.008	4	0.002/4	0.5	SYN	81.29 (83.81; −2.52)	SYN
*C. kefyr*	V2107534	0.001	1	0.0005/1	0.5625	IND	59.37 (59.97; −0.60)	SYN
*C. kefyr*	V2108462	0.004	16	0.001/16	0.5	SYN	91.47 (97.01; −5.54)	SYN

FICI, fractional inhibitory concentration index; INTPN, interpretation; SYN, synergy; IND, no interaction; ISA, isavuconazole; COL, colistin; ATCC, American Type Culture Collection; DSM, Deutsche Sammlung von Mikroorganismen und Zellkulturen; MIC, minimum inhibitory concentration.

**Table 2:** Summary of the *in vitro* interactions of isavuconazole with colistin against common *Candida* species evaluated by EUCAST broth microdilution checkerboard methodology and interpretation by fractional inhibitory concentration index and response surface analysis.

Species (strains), interpretation model	% of strains with the following interaction		
	Synergy	Indifference	Antagonism
*Candida albicans* (10), FICI	0	100	0
*C. albicans* (10), RSA	0	100	0
*Candida glabrata* (10), FICI	90	10	0
*C. glabrata* (10), RSA	10	90	0
*Candida krusei* (10) FICI	80	20	0
*C. krusei* (10), RSA	50	50	0
*Candida parapsilosis* (9), FICI	33	67	0
*C. parapsilosis* (9), RSA	0	100	0
*Candida tropicalis* (10), FICI	90	10	0
*C. tropicalis* (10), RSA	50	50	0
*Candida kefyr* (8), FICI	50	50	0
*C. kefyr* (8), RSA	63	37	0

FICI, fractional inhibitory concentration index; RSA, response surface analysis; EUCAST, European Committee on Antimicrobial Susceptibility Testing.

The 57 *Candida* strains exhibited MICs for isavuconazole ranging from 0.0001 to 0.5 μg/ml (**Table 1**) with MIC50, MIC90, and geometric mean MIC of 0.008, 0.125, and 0.018 μg/ml, respectively. Isavuconazole MICs ranged from 0.0005 to 0.004, 0.125 to 0.5, 0.03 to 0.25, 0.008 to 0.03, 0.008 to 0.06, and 0.0001 to 0.008 µg/ml for *C. albicans*, *C. glabrata*, *C. krusei*, *C. parapsilosis*, *C. tropicalis*, and *C. kefyr*, respectively. When tested alone, colistin exhibited MICs ranging from 16 to 128 µg/ml for the different species (128 μg/ml being the high-off scale MIC) with MIC50, MIC90, and geometric mean MIC of 64, 128, and 63.23 μg/ml, respectively. The best activity of colistin was seen against *C. tropicalis* with MICs ranging from 16 to 32 µg/ml. Significant activity was also seen for *C. kefyr* with a geometric mean MIC of 32 µg/ml. Against *C. krusei*, colistin MICs were high with a geometric mean MIC of 59.71 µg/ml. Colistin showed almost no activity against *C. albicans*, *C. glabrata*, and *C. parapsilosis* with geometric mean MICs of 119.43, 119.43, and 109.73 µg/ml, respectively. The geometric mean MIC for colistin in combination with the synergistic strains was 9.93 µg/ml. Between experiments, isavuconazole and colistin MICs were within ±1 log_2_ dilutions in 99.12% of the cases for all *Candida* species tested (data not shown). The interaction was synergistic for 58% of the strains with FICIs ranging from 0.25 to 0.5 with a geometric mean FICI of 0.4. Synergy was obtained for 0%, 33%, 50%, 80%, 90%, and 90% of *C. albicans*, *C. parapsilosis*, *C. kefyr*, *C. krusei*, *C. glabrata*, and *C. tropicalis*, respectively (**Table 2**). All other interactions were indifferent. The geometric mean FICI for all strains was 0.48.

Analysis of the checkerboard data of the 57 *Candida* strains by the response surface approach led to similar results compared to the FICI results. Although synergy was less frequently obtained than by FICI analysis, the tendency if the combination is synergistic, or for a certain species indifferent (at least 50% of the strains for both interpretation models), was correct in 5/6 cases (**Table 2**). The SUM-SYN-ANT metric for the synergistic strains ranged from 59.37 to 122.67, with a mean of 78.22. For *C. glabrata*, the mean of the SUM-SYN-ANT metric of all strains was 40.42. Synergy was obtained for 0%, 0%, 10%, 50%, 50%, and 67% of *C. albicans*, *C. parapsilosis*, *C. glabrata*, *C. krusei*, *C. tropicalis*, and *C. kefyr*, respectively. When comparing the results of the FICI with the response surface approach, synergy was obtained for at least 50% of the strains for both interpretation models for *C. kefyr*, *C. krusei*, and *C. tropicalis*. One difference between the interpretation techniques was that interaction against *C. glabrata* was synergistic (9 of 10 strains) by FICI and indifferent by response surface analysis (9 of 10 strains).

## Discussion

Advantages of drug repurposing are the use of de‐risked compounds, potentially lower development costs, and shorter development timelines (Pushpakom et al., 2019). Repurposed drugs against *Candida* belong to the antihelmintics (quinacrine), cytotoxic agents (doxorubicin and daunorubicin), or 5α-reductase inhibitors (finasteride) and inhibit filamentation (Miro-Canturri et al., 2019). Colistin is an antibiotic with activity against gram-negative bacteria (Lim et al., 2010), but it has been shown to be able to damage the membrane in *C. albicans* (Yousfi et al., 2019) and increase its permeability (Bibi et al., 2021), which makes the molecule an interesting partner to test combinations with antifungals.

Compared to other studies, the MICs of isavuconazole for the different *Candida* species in this study were in the same range as previously reported (Desnos-Ollivier et al., 2019; Jorgensen et al., 2019). MICs for colistin were comparable to those of another study, but apart from *C. albicans*, only one strain of each species has been tested (Zeidler et al., 2013). Compared to our recently published study on the combination of amphotericin B and colistin, colistin MICs were similar (Schwarz et al., 2022).

Combination MICs of colistin ranged from 1 to 32 μg/ml, with a geometric mean MIC for the synergistic strains of 9.93 μg/ml. Peak serum levels of 13 to 32 have been reported in patients with cystic fibrosis (Reed et al., 2001), which would be sufficient against the strains tested in this study, but colistin use in patients is limited due to its nephrotoxicity (Javan et al., 2015). However, in patients with cryptococcosis, it has been shown that synergy can be achieved with lower serum levels than those tested *in vitro* (Kontoyiannis et al., 2008).

Against common *Candida* species, *in vitro* synergy of combinations including colistin has been reported for echinocandins (Zeidler et al., 2013) and for amphotericin B (Schwarz et al., 2022). Combinations of colistin with azoles were evaluated by two studies. One study found indifference for the combinations of colistin with either fluconazole or itraconazole against *C. albicans*, but both combinations were only tested against one strain (Yousfi et al., 2019). Our study is in accordance with these results. We found indifference for the combination of isavuconazole with colistin by checkerboard and interpretation of the results by FICI and response surface analysis. Another study also evaluated the combination of colistin with fluconazole by checkerboard against one *C. albicans* strain and found synergy *in vitro* and *in vivo* in a *Galleria mellonella* model of invasive candidiasis (Bibi et al., 2021). This discrepancy compared to our results might be strain specific, as only one strain was tested or might be related to the different azole used.

For the other species tested in this study, we found synergy for the majority, or at least for half of the tested strains, for *C. glabrata*, *C. krusei*, *C. tropicalis*, and *C. kefyr*. Only *C. parapsilosis* indifference was found for the majority of the strains. Although synergy was less frequently seen, when the checkboard data were evaluated by response surface analysis, at least 50% of the strains exhibited synergy regardless of the interpretation model used for *C. kefyr*, *C. krusei*, and *C. tropicalis*. For *C. parapsilosis*, both interpretation models evaluated indifference. Between the two interpretation techniques, one difference was found for *C. glabrata*. Interpretation by the FICI showed synergy for 90% of the strains, while interpretation by response surface analysis exhibited indifference for 90% of the strains. Nevertheless, despite the formal indifference of the *C. glabrata* results by response surface analysis, the mean of the SUM-SYN-ANT metric of all strains was quite high (40.42). The discrepancy could indeed be related to the stringent threshold (56.0) used in the present work compared to previous studies (Schwarz et al., 2020a; Schwarz et al., 2022).

In conclusion, we found *in vitro* synergy of the combination of isavuconazole with colistin against *C. glabrata*, *C. krusei*, *C. tropicalis*, and *C. kefyr*. Against *C. albicans* and *C. parapsilosis*, the combination only exhibited indifference. Antagonism was never seen regardless of interpretation model used. These results warrant further animal experiments.

## Data Availability Statement

The datasets presented in this study can be found in online repositories. The names of the repository/repositories and accession number(s) can be found below: https://www.ncbi.nlm.nih.gov/genbank/, OM859334 to OM859357 https://www.ncbi.nlm.nih.gov/genbank/, OL351325 to OL351356.

## Author Contributions

PS wrote the first draft of the manuscript. PS and IN carried out the experiments. PS, IN, A-LB, and ED performed the analysis of the results. ED, PS, GB, and FS contributed to the revisions. All authors have read and agreed to publish the final version of the manuscript.

## Funding

Open Access funding provided by the Open Acess Publication Fund of Philipps-Universität Marburg with support of the Deutsche Forschungsgemeinschaft (DFG, German Research Foundation). This study was supported by internal funding.

## Conflict of Interest

PS has received research grants from Basilea Pharmaceutica, Gilead, and Pfizer; travel grants from Gilead and Pfizer; and speaking fees from Pfizer. During the past 5 years, ED has received research grants from MSD and Gilead; travel grants from Gilead, MSD, Pfizer, and Astellas; and speaker’s fees from Gilead, MSD, and Astellas.

The remaining authors declare that the research was conducted in the absence of any commercial or financial relationships that could be construed as a potential conflict of interest.

## Publisher’s Note

All claims expressed in this article are solely those of the authors and do not necessarily represent those of their affiliated organizations, or those of the publisher, the editors and the reviewers. Any product that may be evaluated in this article, or claim that may be made by its manufacturer, is not guaranteed or endorsed by the publisher.

## References

[B1] Aldejohann A. M. Herz M. Martin R. Walther G. Kurzai O. (2021). Emergence of Resistant *Candida Glabrata* in Germany. JAC Antimicrob. Resist. 3 (3), dlab122. doi: 10.1093/jacamr/dlab122 34377983 PMC8346698

[B2] Arendrup M. C. Meletiadis J. Mouton J. W. Lagrou K. Hamal P. Guinea J. (2020). Method for the Determination of Broth Dilution Minimum Inhibitory Concentrations of Antifungal Agents for Yeasts (EUCAST Definitive Document E.Def 7.3.2), Copenhagen, Denmark.

[B3] Bibi M. Murphy S. Benhamou R. I. Rosenberg A. Ulman A. Bicanic T.. (2021). Combining Colistin and Fluconazole Synergistically Increases Fungal Membrane Permeability and Antifungal Cidality. ACS Infect. Dis. 7 (2), 377–389. doi: 10.1021/acsinfecdis.0c00721 33471513 PMC7887753

[B4] Bidaud A. L. Schwarz P. Herbreteau G. Dannaoui E. (2021). Techniques for the Assessment of *In Vitro* and *In Vivo* Antifungal Combinations. J. Fungi (Basel) 7 (2), 113. doi: 10.3390/jof7020113 33557026 PMC7913650

[B5] Cornely O. A. Bassetti M. Calandra T. Garbino J. Kullberg B. J. Lortholary O.. (2012). ESCMID Guideline for the Diagnosis and Management of Candida Diseases 2012: non-Neutropenic Adult Patients. Clin. Microbiol. Infect. 18 Suppl 7, 19–37. doi: 10.1111/1469-0691.12039 23137135

[B6] Day J. N. Chau T. T. H. Wolbers M. Mai P. P. Dung N. T. Mai N. H.. (2013). Combination Antifungal Therapy for Cryptococcal Meningitis. N Engl. J. Med. 368 (14), 1291–1302. doi: 10.1056/NEJMoa1110404 23550668 PMC3978204

[B7] Desnos-Ollivier M. Bretagne S. Boullie A. Gautier C. Dromer F. Lortholary O.. (2019). Isavuconazole MIC Distribution of 29 Yeast Species Responsible for Invasive Infections, (2015-2017). Clin. Microbiol. Infect. 25 (5), 634.e631–634.e634. doi: 10.1016/j.cmi.2019.02.007 30771532

[B8] Di Veroli G. Y. Fornari C. Wang D. Mollard S. Bramhall J. L. Richards F. M.. (2016). Combenefit: An Interactive Platform for the Analysis and Visualization of Drug Combinations. Bioinformatics 32 (18), 2866–2868. doi: 10.1093/bioinformatics/btw230 27153664 PMC5018366

[B9] Javan A. O. Shokouhi S. Sahraei Z. (2015). A Review on Colistin Nephrotoxicity. Eur. J. Clin. Pharmacol. 71 (7), 801–810. doi: 10.1007/s00228-015-1865-4 26008213

[B10] Johnson M. D. MacDougall C. Ostrosky-Zeichner L. Perfect J. R. Rex J. H. (2004). Combination Antifungal Therapy. Antimicrob. Agents Chemother. 48 (3), 693–715. doi: 10.1128/aac.48.3.693-715.2004 14982754 PMC353116

[B11] Jorgensen K. M. Astvad K. M. T. Hare R. K. Arendrup M. C. (2019). EUCAST Susceptibility Testing of Isavuconazole: MIC Data for Contemporary Clinical Mold and Yeast Isolates. Antimicrob. Agents Chemother. 63 (6):e00073-19. doi: 10.1128/AAC.00073-19 30910898 PMC6535523

[B12] Kontoyiannis D. P. Lewis R. E. Alexander B. D. Lortholary O. Dromer F. Gupta K. L.. (2008). Calcineurin Inhibitor Agents Interact Synergistically With Antifungal Agents *In Vitro* Against *Cryptococcus Neoformans* Isolates: Correlation With Outcome in Solid Organ Transplant Recipients With Cryptococcosis. Antimicrob. Agents Chemother. 52 (2), 735–738. doi: 10.1128/AAC.00990-07 18070977 PMC2224743

[B13] Ledoux M. P. Denis J. Nivoix Y. Herbrecht R. (2018). Isavuconazole: A New Broad-Spectrum Azole. Part 2: Pharmacokinetics and Clinical Activity. J. Mycol. Med. 28 (1), 15–22. doi: 10.1016/j.mycmed.2018.02.002 29551442

[B14] Lim L. M. Ly N. Anderson D. Yang J. C. Macander L. Jarkowski A. 3rd. (2010). Resurgence of Colistin: A Review of Resistance, Toxicity, Pharmacodynamics, and Dosing. Pharmacotherapy 30 (12), 1279–1291. doi: 10.1592/phco.30.12.1279 21114395 PMC4410713

[B15] Miro-Canturri A. Ayerbe-Algaba R. Smani Y. (2019). Drug Repurposing for the Treatment of Bacterial and Fungal Infections. Front. Microbiol. 10. doi: 10.3389/fmicb.2019.00041 PMC6360151 30745898

[B16] Mohr A. Simon M. Joha T. Hanses F. Salzberger B. Hitzenbichler F. (2020). Epidemiology of Candidemia and Impact of Infectious Disease Consultation on Survival and Care. Infection 48 (2), 275–284. doi: 10.1007/s15010-020-01393-9 32052287

[B17] Montagna M. T. Caggiano G. Lovero G. De Giglio O. Coretti C. Cuna T.. (2013). Epidemiology of Invasive Fungal Infections in the Intensive Care Unit: Results of a Multicenter Italian Survey (AURORA Project). Infection 41 (3), 645–653. doi: 10.1007/s15010-013-0432-0 23463186 PMC3671106

[B18] Odds F. C. (2003). Synergy, Antagonism, and What the Chequerboard Puts Between Them. J. Antimicrob. Chemother. 52 (1), 1. doi: 10.1093/jac/dkg301 12805255

[B19] Orozco A. S. Higginbotham L. M. Hitchcock C. A. Parkinson T. Falconer D. Ibrahim A. S.. (1998). Mechanism of Fluconazole Resistance in *Candida Krusei* . Antimicrob. Agents Chemother. 42 (10), 2645–2649. doi: 10.1128/AAC.42.10.2645 9756770 PMC105912

[B20] Pfaller M. A. Boyken L. Hollis R. J. Messer S. A. Tendolkar S. Diekema D. J. (2006). *In Vitro* Susceptibilities of *Candida* spp. To Caspofungin: Four Years of Global Surveillance. J. Clin. Microbiol. 44 (3), 760–763. doi: 10.1128/JCM.44.3.760-763.2006 16517851 PMC1393154

[B21] Pfaller M. A. Diekema D. J. Gibbs D. L. Newell V. A. Ellis D. Tullio V.. (2010). Results From the ARTEMIS DISK Global Antifungal Surveillance Study 1997 to 2007: A 10.5-Year Analysis of Susceptibilities of *Candida* Species to Fluconazole and Voriconazole as Determined by CLSI Standardized Disk Diffusion. J. Clin. Microbiol. 48 (4), 1366–1377. doi: 10.1128/JCM.02117-09 20164282 PMC2849609

[B22] Pfaller M. Neofytos D. Diekema D. Azie N. Meier-Kriesche H. U. Quan S. P.. (2012). Epidemiology and Outcomes of Candidemia in 3648 Patients: Data From the Prospective Antifungal Therapy (PATH Alliance(R)) Registry 2004-2008. Diagn. Microbiol. Infect. Dis. 74 (4), 323–331. doi: 10.1016/j.diagmicrobio.2012.10.003 23102556

[B23] Pilmis B. Yang Z. Lanternier F. Lortholary O. (2017). “Systemic Candidiasis,” in Infectious Diseases. Eds. Cohen J. Powderly W. G. Opal S. M. (Amsterdam: Elsevier), 439–445.

[B24] Pushpakom S. Iorio F. Eyers P. A. Escott K. J. Hopper S. Wells A.. (2019). Drug Repurposing: Progress, Challenges and Recommendations. Nat. Rev. Drug Discovery 18 (1), 41–58. doi: 10.1038/nrd.2018.168 30310233

[B25] Reed M. D. Stern R. C. O'Riordan M. A. Blumer J. L. (2001). The Pharmacokinetics of Colistin in Patients With Cystic Fibrosis. J. Clin. Pharmacol. 41 (6), 645–654. doi: 10.1177/00912700122010537 11402633

[B26] Schöneweck F. Schmitz R. P. H. Rissner F. Scherag A. Loffler B. Pletz M. W.. (2021). The Epidemiology of Bloodstream Infections and Antimicrobial Susceptibility Patterns in Thuringia, Germany: A Five-Year Prospective, State-Wide Surveillance Study (AlertsNet). Antimicrob. Resist. Infect. Control 10 (1), 132. doi: 10.1186/s13756-021-00997-6 34493334 PMC8424790

[B27] Schroeder M. Weber T. Denker T. Winterland S. Wichmann D. Rohde H.. (2020). Epidemiology, Clinical Characteristics, and Outcome of Candidemia in Critically Ill Patients in Germany: A Single-Center Retrospective 10-Year Analysis. Ann. Intensive Care 10 (1), 142. doi: 10.1186/s13613-020-00755-8 33064220 PMC7567770

[B28] Schwarz P. Bidaud A. L. Dannaoui E. (2020a). *In Vitro* Synergy of Isavuconazole in Combination With Colistin Against *Candida Auris* . Sci. Rep. 10 (1), 21448. doi: 10.1038/s41598-020-78588-5 33293607 PMC7722718

[B29] Schwarz P. Bretagne S. Gantier J. C. Garcia-Hermoso D. Lortholary O. Dromer F.. (2006a). Molecular Identification of Zygomycetes From Culture and Experimentally Infected Tissues. J. Clin. Microbiol. 44 (2), 340–349. doi: 10.1128/JCM.44.2.340-349.2006 16455881 PMC1392659

[B30] Schwarz P. Cornely O. A. Dannaoui E. (2019). Antifungal Combinations in Mucorales: A Microbiological Perspective. Mycoses. 62 (9), 746–60. doi: 10.1111/myc.12909 30830980

[B31] Schwarz P. Djenontin E. Dannaoui E. (2020b). Colistin and Isavuconazole Interact Synergistically *In Vitro* Against *Aspergillus Nidulans* and *Aspergillus Niger* . Microorganisms 8 (9), 1447. doi: 10.3390/microorganisms8091447 PMC7564879 32967270

[B32] Schwarz P. Dromer F. Lortholary O. Dannaoui E. (2006b). Efficacy of Amphotericin B in Combination With Flucytosine Against Flucytosine-Susceptible or Flucytosine-Resistant Isolates of *Cryptococcus Neoformans* During Disseminated Murine Cryptococcosis. Antimicrob. Agents Chemother. 50 (1), 113–120. doi: 10.1128/AAC.50.1.113-120.2006 16377675 PMC1346792

[B33] Schwarz P. Janbon G. Dromer F. Lortholary O. Dannaoui E. (2007). Combination of Amphotericin B With Flucytosine is Active *In Vitro* Against Flucytosine-Resistant Isolates of *Cryptococcus Neoformans* . Antimicrob. Agents Chemother. 51 (1), 383–385. doi: 10.1128/AAC.00446-06 17043122 PMC1797681

[B34] Schwarz P. Nikolskiy I. Bidaud A. L. Sommer F. Bange G. Dannaoui E. (2022). *In Vitro* Activity of Amphotericin B in Combination With Colistin Against Fungi Responsible for Invasive Infections. J. Fungi (Basel) 8 (2), 115. doi: 10.3390/jof8020115 35205869 PMC8880464

[B35] Vannini M. Emery S. Lieutier-Colas F. Legueult K. Mondain V. Retur N.. (2022). Epidemiology of Candidemia in NICE Area, France: A Five-Year Study of Antifungal Susceptibility and Mortality. J. Mycol. Med. 32 (1), 101210. doi: 10.1016/j.mycmed.2021.101210 34768155

[B36] Vitale R. G. (2021). Role of Antifungal Combinations in Difficult to Treat *Candida* Infections. J. Fungi (Basel) 7 (9), 731. doi: 10.3390/jof7090731 34575770 PMC8468556

[B37] Yapar N. (2014). Epidemiology and Risk Factors for Invasive Candidiasis. Ther. Clin. Risk Manag. 10, 95–105. doi: 10.2147/TCRM.S40160 24611015 PMC3928396

[B38] Yousfi H. Ranque S. Rolain J. M. Bittar F. (2019). *In Vitro* Polymyxin Activity Against Clinical Multidrug-Resistant Fungi. Antimicrob. Resist. Infect. Control 8, 66. doi: 10.1186/s13756-019-0521-7 31044071 PMC6480676

[B39] Zeidler U. Bougnoux M. E. Lupan A. Helynck O. Doyen A. Garcia Z.. (2013). Synergy of the Antibiotic Colistin With Echinocandin Antifungals in *Candida* Species. J. Antimicrob. Chemother. 68 (6), 1285–1296. doi: 10.1093/jac/dks538 23378416

[B40] Zhao Y. Tsang C. C. Xiao M. Cheng J. Xu Y. Lau S. K.. (2015). Intra-Genomic Internal Transcribed Spacer Region Sequence Heterogeneity and Molecular Diagnosis in Clinical Microbiology. Int. J. Mol. Sci. 16 (10), 25067–25079. doi: 10.3390/ijms161025067 26506340 PMC4632790

